# Supplementary figures and images for: Social Learning of a Spatial Task by Observation Alone
Source: Front Behav Neurosci. 2022 Jul 13;16:902675. doi: 10.3389/fnbeh.2022.902675 (PMC9325960; doi:10.3389/fnbeh.2022.902675)

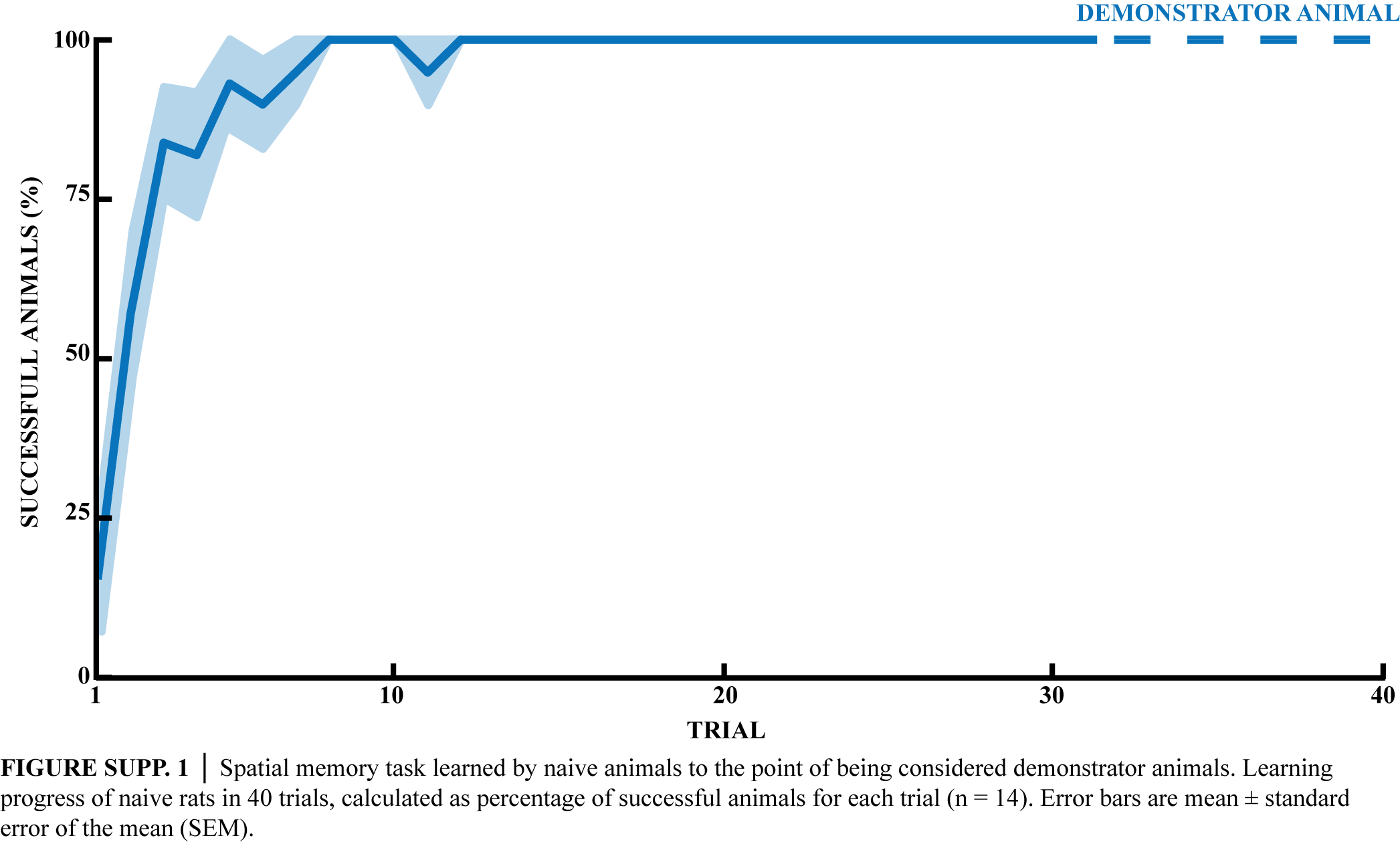

Supplement: Supplementary Figure 1 — Spatial memory task learned by naive animals to the point of being considered demonstrator animals. Learning progress of naive rats in 40 trials, calculated as the percentage of successful animals for each trial (n = 14). Error bars are mean ± standard error of the mean (SEM). [file Image_1.TIF]
