# Supplementary material for: Social Learning of a Spatial Task by Observation Alone
Source: Front Behav Neurosci. 2022 Jul 13;16:902675. doi: 10.3389/fnbeh.2022.902675 (PMC9325960; doi:10.3389/fnbeh.2022.902675)
Supplement: Supplementary Table 1 — Total number of animals per group. Note that animals that spent 2 h without successfully finding the reward were viewed as non-performers (one observer, two naïve rats) and discarded. In addition, all rats showing problems in the video recordings were removed from the count during the time of the problem because the evaluation could not be double-checked (only naïve rats during the first session). [file Table_1.pdf]

Naïve	Observer	Demonstrator
**25**	**19**	**14**
Rewarded	Rewarded	
18	6	
non rewarded	non rewarded	
7	8	
	CPP non-rewarded	
	5	

TABLE SUPP. 1 | Total number of animals per group. Note that animals that spent 2 hours without successfully finding the reward were viewed as non-performers (1 observer, 2 naïve rats) and discarded. In addition, all rats showing problems in the video recordings were removed from the count during the time of the problem because the evaluation could not be double-checked (only naïve rats during first session).
