# Supplementary material for: Social Learning of a Spatial Task by Observation Alone
Source: Front Behav Neurosci. 2022 Jul 13;16:902675. doi: 10.3389/fnbeh.2022.902675 (PMC9325960; doi:10.3389/fnbeh.2022.902675)
Supplement: Supplementary Table 2 — Summary of results for each rewarded group. [file Table_2.pdf]

Time to success for rewarded animals (s)			
Trials	Naïve (n=18)	Observer (n=6)	Demonstrator (n=14)
1	1515.6	344.7	200.3
2	277.3	92.3	43.3
3	347.6	212.0	10.9
4	64.9	40.3	8.2
5	110.9	49.5	6.8

Percentage of success for rewarded animals (%)			
Trials	Naïve (n=18)	Observer (n=6)	Demonstrator (n=14)
1	12.5	100.0	100.0
2	55.6	100.0	100.0
3	83.3	100.0	100.0
4	81.3	100.0	100.0
5	92.9	100.0	100.0
6	89.5	100.0	100.0
7	94.7	100.0	100.0
8	100.0	100.0	100.0
9	100.0	100.0	100.0
10	100.0	100.0	100.0
11	94.7	100.0	100.0
12	100.0	100.0	100.0
13	100.0	100.0	100.0
14	100.0	100.0	100.0
15	100.0	100.0	100.0

Mean number of mistakes for rewarded animals			
Trials	Naïve (n=18)	Observer (n=6)	Demonstrator (n=14)
1	2.0	0.0	0.0
2	0.7	0.0	0.0
3	0.2	0.0	0.0
4	0.2	0.0	0.0
5	0.1	0.0	0.0
6	0.1	0.0	0.0
7	0.2	0.0	0.0
8	0.0	0.0	0.0
9	0.0	0.0	0.0
10	0.0	0.0	0.0
11	0.1	0.0	0.0
12	0.0	0.0	0.0
13	0.0	0.0	0.0
14	0.0	0.0	0.0
15	0.0	0.0	0.0

TABLE SUPP. 2 | Summary of results for each rewarded group.
